# Checking Genetic Homogeneity Between Two Samples Using Summary Statistics With Application to Mendelian Randomization

**DOI:** 10.1002/sim.70663

**Published:** 2026-07-07

**Authors:** Kai Wang, Grace Z. Wang

**Affiliations:** ^1^ Department of Biostatistics University of Iowa Iowa City Iowa USA; ^2^ Department of Biostatistics Harvard T.H. Chan School of Public Health Boston Massachusetts USA

**Keywords:** genetic homogeneity, GWAS, linear regression, Mendelian randomization, summary statistics

## Abstract

**Background:**

A common assumption in two‐sample summary‐data Mendelian randomization (MR) studies is that the effect of an instrumental single nucleotide polymorphism (SNP) on both the exposure and the outcome is consistent across the exposure and outcome samples. This assumption is more likely to hold when the minor allele frequency (MAF) of the instrumental SNP is similar in the two samples. However, in the common situation where MAF information is unavailable, there is currently no formal method to assess this condition, apart from heuristically matching the ethnic backgrounds of the exposure and outcome samples.

**Method:**

We propose a simple method to assess whether the genetic variance of a SNP, which is uniquely determined by its minor allele frequency (MAF) under Hardy–Weinberg equilibrium, is the same across two genome‐wide association studies (GWASs). The method is motivated by the observation that, in linear‐model GWASs, although the absolute magnitude of a SNP's variance cannot be recovered from summary statistics, its variance relative to that of other SNPs can be inferred. This observation continues to hold approximately when the underlying model is nonlinear.

**Results:**

We propose a V‐V plot and a modified Bland–Altman plot to identify SNPs that have different genetic variances between two GWASs. Some published two‐sample summary‐data MR studies, unfortunately, seem to include SNPs that are not genetically homogeneous.

**Conclusions:**

The proposed method provides a useful tool for enhancing the quality of a two‐sample summary‐data MR study. We advocate checking for equality of SNP MAFs between the exposure and the outcome GWASs before conducting a two‐sample summary‐data MR analysis, and our proposed method serves this purpose when the MAF information is missing in at least one of the two GWASs.

## 1 Introduction

Mendelian randomization (MR) is a popular application of the instrumental variable (IV) method [1, 2]. Its aim is to estimate the causal effect of an exposure on an outcome while controlling for unobserved confounding factors. Its popularity is partly due to its simplicity: it can be conducted using only summary statistics from a genome‐wide association study (GWAS) on the exposure and summary statistics from a GWAS on the outcome and these two GWASs can be based on two different samples [3]. Indeed, using summary‐level data that come from different samples is common in MR analysis [4, 5].

Although two‐sample summary‐data MR offers practical advantages, it presents distinct methodological challenges. The validity of such analyses hinges on several critical assumptions. Chief among them is the expectation that the effect size of an instrumental Single nucleotide polymorphisms (SNP) on both the exposure and the outcome remains consistent across the respective GWAS samples [5, 6]. While this assumption may hold in certain contexts, it becomes problematic in the presence of genetic heterogeneity [5, 6]. Specifically, locus or allelic heterogeneity can undermine this assumption [7]. To bolster its credibility, one can examine whether the MAF of instrumental SNPs is comparable between the exposure and outcome GWASs. Consistency in MAF across samples helps mitigate the risk of heterogeneity and strengthens the validity of the MR analysis.

However, in data analysis, MAFs are typically not checked for their equality even when their information is available in both GWASs. This neglect might be due to the fact that the MAF information is often missing in the summary statistics for a GWAS. In practice, it is common to satisfy this requirement by matching the ethnicity of the populations underlying the two GWASs. For instance, both the exposure GWAS and the outcome GWAS are required to be conducted on samples from the same population. Such a heuristic argument appears to make sense. However, as demonstrated later using real data examples, there are SNPs that do not seem to share the same MAF between two GWASs that are ethnicity‐matched. Sometimes the reported MAFs in the summary statistics of a GWAS may be from a reference panel, such as the UK Biobank, and may be biased. Clearly, SNPs with different MAFs in the exposure GWAS and the outcome GWAS are not intended to be included in an MR analysis. Given the popularity of two‐sample summary‐data MR, there is an urgent need for methods that address this issue.

We propose a V‐V plot and a modified Bland–Altman plot to identify SNPs that have different genetic variances between two GWASs. Assuming that the Hardy‐Weinberg equilibrium holds in two samples, the genetic variance of a SNP in one sample is the same as that in another sample if and only if its MAFs in these two samples are the same. Hence these plots can be used to identify SNPs that have different MAFs between two samples. Since the genetic variance is invariant to the choice of the reference allele, this method does not rely on whether the reference alleles used for these two samples are the same. This method is motivated by a simple identity for a linear model GWAS. It holds approximately for nonlinear models, such as a logistic model GWAS.

In this report, “genetic homogeneity” for an instrument SNP refers to the equality of its genetic variance across two samples. Therefore, our proposed method is a method for checking genetic homogeneity. To the best of our knowledge, no such methods exist in the literature that use only summary statistics.

In what follows, we introduce the concepts and assumptions regarding two‐sample summary‐data MR and explain why genetic homogeneity is desired for a valid analysis. Then, we propose the V‐V plot and the modified Bland–Altman plot for checking the genetic homogeneity of a SNP between two samples using summary statistics of the two GWASs. A simulation study is used to demonstrate the proposed method and investigate its ability to detect genetic homogeneity. Finally, its performance is studied using real data examples that are used in some methodology publications as well as some existing original studies, where the goal is to see whether the IV SNPs are genetically homogeneous between the two GWASs.

## 2 Two‐Sample Summary‐Data MR

Denote the exposure by x and the outcome by y. Consider the following linear regression model:

(1) y=bx+u,

where b is the causal effect of x on y and u represents unobserved factors satisfying Cov(x,u)≠0. Without loss of generality, the model variables are centered so that E(y)=E(x)=E(u)=0. Importantly, since Cov(x,u)≠0, the least squares estimate of b is biased and inconsistent. The IV method can be used to generate a consistent estimate of b.

Let z denote the genotype score of an instrument SNP. The genotype score is the number of copies of the reference allele chosen for this SNP. As an instrument SNP, z satisfies three assumptions [8, 9]: (Relevance) It is associated with exposure x (i.e., Cov(z,x)≠0); (Exclusion Restriction) It affects outcome y only through its association with exposure x (i.e., z has no direct effect on y); and (Exchangeability) It is not associated with any confounders of the exposure‐outcome association (i.e., Cov(z,u)=0). Under these assumptions, it is easy to see from Equation (1) that

Cov(z,y)=bCov(z,x).

Therefore, since Cov(z,x)≠0, the causal effect b can be written

(2) $$b=\frac{Cov(z,y)}{Cov(z,x)}=\frac{Cov(z,y)/\mathrm{Var}(z)}{Cov(z,x)/\mathrm{Var}(z)}=\frac{\Gamma }{\gamma },$$

where Γ=Cov(z,y)/Var(z) is the linear regression coefficient of y on z and γ=Cov(z,x)/Var(z) is the linear regression coefficient of x on z. Estimates of the Γ and γ, denoted by $\hat{\Gamma }$ and $\hat{\gamma }$, respectively, and their standard errors (SEs) are available from the exposure GWAS and the outcome GWAS as summary statistics. When $\hat{\Gamma }$ and $\hat{\gamma }$ are consistent for Γ and γ, respectively, $\hat{b}=\hat{\Gamma }/\hat{\gamma }$ will be a consistent estimate for Γ/γ.

In the method to be proposed in this report, the summary statistics also include the sample sizes used for each GWAS. These sample sizes are allowed to be different for different SNPs to accommodate missing values in genotype scores.

The presentation above is for one‐sample MR where Γ and γ are from the same sample. In two‐sample summary‐data MR, the exposure GWAS and the outcome GWAS are typically from two different samples. As a result, the value of Var(z) may not be the same in these two samples, and Equation (2) is unlikely to hold as Γ/γ is no longer equal to Cov(z,y)/Cov(z,x). This phenomenon, due to heterogeneous samples has been noticed elsewhere [5, 6].

The ratio Γ/γ in Equation (2) plays an important role in MR. It forms the foundation for many popular methods for two‐sample summary‐data MR. For instance, the inverse variance weighting (IVW) method [10, 11] and many variations of IVW, such as MR‐Egger regression [12], estimate b by a weighted average of $\hat{\Gamma }/\hat{\gamma }$ at all the instrumental SNPs and the weighted median estimator uses the median of a weight‐adjusted distribution [13]. If any one of the instrument SNPs is not genetically homogeneous, results from these methods for two‐sample summary‐data MR will be in question.

In the next section, we propose a graphical approach to detect SNPs that are not homogeneous between two samples.

## 3 V‐V Plot and Modified Bland–Altman Plot

We first introduce a general result regarding a linear model GWAS and then use this result to construct two plots for checking the genetic homogeneity of a SNP between two GWASs. Although these plots originate from a linear model GWAS, they work approximately for nonlinear model GWASs, such as logistic model GWASs or Poisson model GWASs.

### 3.1 A General Result Regarding a Linear Regression GWAS

Consider a linear model GWAS on a trait w where the number of SNPs is m and the number of covariates is k. Without loss of generality, it is assumed that the effect of the k covariates has been controlled for and w is the residual after removing the effect of these k covariates. w is further centered in order to remove the effect of the intercept term. In the context of two‐sample summary‐data MR, w can be the (covariate‐corrected) exposure x or outcome y. For the jth SNP, j=1,…,m, define $P_{j}=z_{j}{\left(z_{j}^{\prime }z_{j}\right)}^{-1}z_{j}^{\prime }$ where $z_{j}$ is a column vector of centered genotype scores. The estimate of the (marginal) effect of the jth SNP on w is denoted by ${\hat{\beta }}_{j}$, which is usually reported by a GWAS.

Using standard results on a ordinary least squares method for a linear regression, the residual variance is estimated by $w^{\prime }(I-P_{j})w/(n_{j}-2-k)$, where $n_{j}$ the number of genotyped subjects at the jth SNP, w is a vector of w's at the $n_{j}$ genotyped subjects, and I is an identity matrix. Furthermore,

$$\begin{array}{cc} \mathrm{Var}({\hat{\beta }}_{j}) & =\frac{w^{\prime }(I-P_{j})w}{(n_{j}-2-k)\cdot z_{j}^{\prime }z_{j}}\;\text{and}\\ {\hat{\beta }}_{j}^{2}\cdot z_{j}^{\prime }z_{j} & =w^{\prime }P_{j}w,\;\;\;j=1,\ldots ,m. \end{array}$$

Therefore,

$$w^{\prime }w=w^{\prime }(I-P_{j})w+w^{\prime }P_{j}w=\frac{z_{j}^{\prime }z_{j}}{v_{j}},\;\;\;j=1,\ldots ,m,$$

where

(3) $$v_{j}=\frac{z_{j}^{\prime }z_{j}}{w^{\prime }w}=\frac{\hat{\mathrm{Var}}(z_{j})}{\hat{\mathrm{Var}}(w)}=\frac{1}{{\hat{\beta }}_{j}^{2}+(n_{j}-2-k)\mathrm{Var}({\hat{\beta }}_{j})},\;\;\;j=1,\ldots ,m.$$

Here $\hat{\mathrm{Var}}(\cdot )$ denotes the sample variance of a variable. Clearly, $v_{j}$ is the ratio of the sample genotypic variance of SNP j to the sample variance of w. It is straightforward to calculate $v_{j}$ from summary statistics ${\hat{\beta }}_{j}$, $SE({\hat{\beta }}_{j})$, and $n_{j}$ using Equation (3).

Equation (3) holds exactly for a linear model GWAS. For a logistic model GWAS, it holds approximately up to a multiplying constant. A proof is provided in the Appendix. Similar results hold for other generalized linear models, such as Poison regression model for counts.

### 3.2 V‐V Plot and Modified Bland–Altman Plot

Let $v_{j}^{\left(x\right)}$ denote the value of $v_{j}$ defined in Equation (3) computed for the exposure GWAS and $v_{j}^{\left(y\right)}$ for the outcome GWAS. These two GWASs can have different sample sizes and a different number of covariates. As mentioned previously, $v_{j}^{\left(x\right)}$ is the ratio of the sample variance of the genotype score for SNP j to the sample variance of x and $v_{j}^{\left(y\right)}$ has a similar interpretation. That is, both $v_{j}^{\left(x\right)}$ and $v_{j}^{\left(y\right)}$ depend on the unknown quantities $\hat{\mathrm{Var}}(x)$ and $\hat{\mathrm{Var}}(y)$, respectively. To remove such dependence, we consider the relative ratios of $v_{j}^{\left(x\right)}$ and $v_{j}^{\left(y\right)}$ to their respective means. Define

$${\dot{v}}_{j}^{\left(x\right)}=\frac{v_{j}^{\left(x\right)}}{m^{-1}\sum_{j}v_{j}^{\left(x\right)}},\;\;{\dot{v}}_{j}^{\left(y\right)}=\frac{v_{j}^{\left(y\right)}}{m^{-1}\sum_{j}v_{j}^{\left(y\right)}},\;\;j=1,\ldots ,m.$$

Then from Equation (3),

$$\begin{array}{cc} {\dot{v}}_{j}^{\left(x\right)} & =\frac{\hat{\mathrm{Var}}{\left(z_{j}\right)}^{\left(x\right)}}{m^{-1}\overset{m}{\underset{j=1}{\sum }}\hat{\mathrm{Var}}{\left(z_{j}\right)}^{\left(x\right)}},\\ \;\;{\dot{v}}_{j}^{\left(y\right)} & =\frac{\hat{\mathrm{Var}}{\left(z_{j}\right)}^{\left(y\right)}}{m^{-1}\overset{m}{\underset{j=1}{\sum }}\hat{\mathrm{Var}}{\left(z_{j}\right)}^{\left(y\right)}},\;\;j=1,\ldots ,m, \end{array}$$

where $\hat{\mathrm{Var}}{\left(z_{j}\right)}^{\left(x\right)}$ and $\hat{\mathrm{Var}}{\left(z_{j}\right)}^{\left(y\right)}$ are the sample variances of the jth SNP score in the exposure GWAS and the outcome GWAS, respectively. The right‐hand‐sides of the above two equations depend on only $\hat{\mathrm{Var}}{\left(z_{j}\right)}^{\left(x\right)}$ and $\hat{\mathrm{Var}}{\left(z_{j}\right)}^{\left(y\right)}$, j=1,…,m. ${\dot{v}}_{j}^{\left(x\right)}$ and ${\dot{v}}_{j}^{\left(y\right)}$ are normalized version of $v_{j}^{\left(x\right)}$ and $v_{j}^{\left(y\right)}$, respectively. That is, although $v_{j}^{\left(x\right)}$ and $v_{j}^{\left(y\right)}$ can not be calculated from the summary statistics, ${\dot{v}}_{j}^{\left(x\right)}$ and ${\dot{v}}_{j}^{\left(y\right)}$ can due to the following relationships:

$$\overset{m}{\underset{j=1}{\sum }}{\dot{v}}_{j}^{\left(x\right)}=\overset{m}{\underset{j=1}{\sum }}{\dot{v}}_{j}^{\left(y\right)}=m.$$

The set $\left\{{\dot{v}}_{j}^{\left(x\right)}:j=1,\ldots ,m\right\}$ gives a relative “profile” of $v_{j}^{\left(x\right)}$ and the set $\left\{{\dot{v}}_{j}^{\left(y\right)}:j=1,\ldots ,m\right\}$ is explained the same way for the outcome. We note that

$$\hat{\mathrm{Var}}{\left(z_{j}\right)}^{\left(x\right)}=\hat{var}{\left(z_{j}\right)}^{\left(y\right)}\Leftrightarrow v_{j}^{\left(x\right)}=v_{j}^{\left(y\right)}\Leftrightarrow {\dot{v}}_{j}^{\left(x\right)}={\dot{v}}_{j}^{\left(y\right)},\;\;j=1,\ldots ,m.$$

That is, in order to check whether $\hat{var}{\left(z_{j}\right)}^{\left(x\right)}=\hat{var}{\left(z_{j}\right)}^{\left(y\right)},j=1,\ldots ,m$, it is sufficient to check whether ${\dot{v}}_{j}^{\left(x\right)}={\dot{v}}_{j}^{\left(y\right)},j=1,\ldots ,m$. If the latter is true, a plot of ${\dot{v}}_{j}^{\left(x\right)}$ against ${\dot{v}}_{j}^{\left(y\right)}$ should follow a 45‐degree line with an intercept equal to 0. Deviation from this 45‐degree line is an indication that the SNP is not genetically homogeneous, and it should be excluded from the two‐sample summary‐data MR. If there are a large number of SNPs that are not genetically homogeneous, the two samples may be of different genetic backgrounds.

For simplicity, this plot of with a 45‐degree line going through point (0,0) is named the V‐V plot hereafter. We note that $\left({\dot{v}}_{j}^{\left(x\right)},{\dot{v}}_{j}^{\left(y\right)}\right)$ depends on the whole set of points $\left\{(v_{j}^{\left(x\right)},v_{j}^{\left(y\right)}),j=1,\ldots ,m\right\}$. When the number of SNPs m is small, this dependence may cause a point $\left({\dot{v}}_{j}^{\left(x\right)},{\dot{v}}_{j}^{\left(y\right)}\right)$ to be away from the 45‐degree line even though $v_{j}^{\left(x\right)}=v_{j}^{\left(y\right)}$. However, this is not expected to be an issue when m is large.

Since ${\dot{v}}_{j}^{\left(x\right)}$ and ${\dot{v}}_{j}^{\left(y\right)}$ represent the relative size of $\mathrm{Var}(z_{j})$ in different samples, it is natural to compare them using a Bland–Altman plot. A Bland–Altman plot is equivalent to rotating the V‐V plot clockwise by 45 degrees, so that the horizontal axis corresponds to ${\dot{v}}_{j}^{\left(y\right)}+{\dot{v}}_{j}^{\left(x\right)}$ and the vertical axis corresponds to ${\dot{v}}_{j}^{\left(y\right)}-{\dot{v}}_{j}^{\left(x\right)}$. In the current situation, it is more meaningful to consider the relative size of ${\dot{v}}_{j}^{\left(y\right)}-{\dot{v}}_{j}^{\left(x\right)}$ to ${\dot{v}}_{j}^{\left(y\right)}+{\dot{v}}_{j}^{\left(x\right)}$. Therefore, we consider a modified Bland–Altman plot. Rather than using ${\dot{v}}_{j}^{\left(y\right)}-{\dot{v}}_{j}^{\left(x\right)}$, the vertical axis is

$$\frac{{\dot{v}}_{j}^{\left(y\right)}-{\dot{v}}_{j}^{\left(x\right)}}{{\dot{v}}_{j}^{\left(y\right)}+{\dot{v}}_{j}^{\left(x\right)}}.$$

Its range is (−1,1). A large absolute relative difference implies that the genetic variances of that SNP are different in the two samples.

The SNPs are not required to be independent of each other for either the V‐V plot or the modified Bland–Altman plot.

The proposed V‐V plot and the modified Bland–Altman plot have been implemented in R package iGasso.

### 3.3 Simulation Studies

To demonstrate the utility of the proposed V‐V plot and the modified Bland–Altman plot, we conducted a simulation study using the effect allele frequencies contained in variable eaf.exposure of the bmi.bmi data in the R package mr.raps. This variable contains allele frequencies for 812 SNPs. More details of this data are given in the next section and Table 2.

Three sets of linear regression GWAS summary statistics are simulated. Two of them, referred to as Samples 1 and 2, respectively, share the same allele frequencies, which are set to equal to those in variable eaf.exposure. For the third set of summary statistics, referred to as Sample 3, the allele frequencies are obtained by adding independent Uniform(0.01, 0.05) noise to the MAFs obtained from variable eaf.exposure. Given the allele frequency at a SNP, genotype scores at this SNP are generated for 10 000 subjects independently from a binomial distribution under the Hardy–Weinberg equilibrium. For each sample, the trait for subject i, denoted by $w_{i}$, is set equal to $w_{i}=\sum_{j=1}^{812}b_{j}z_{ij}+e_{i}$, where $b_{j}$s are independently generated from Uniform (−0.2,0.2) and $z_{ij}$ is the genotype score at SNP j, $e_{i}∼N(0,1)$ independently, i=1,…,10000. Summary statistics at SNP j are obtained from a marginal linear regression of $w_{i}$s over $z_{ij}$s in an obvious manner. The averages of the F‐statistics are 10.653, 10.834, and 10.963, respectively, in these three samples, and the averages of the $R^{2}$ statistics are around 0.001 in all three samples.

**FIGURE 1:**
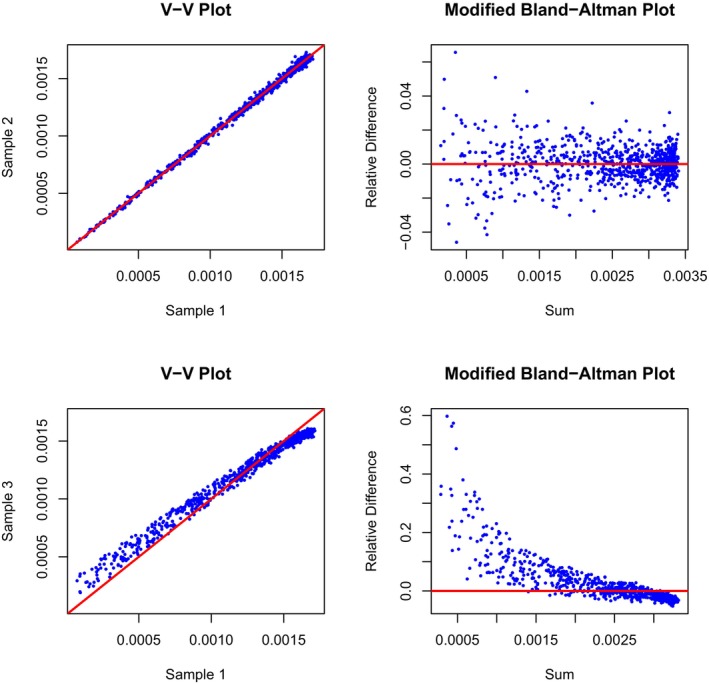
The V‐V plot and the modified Bland–Altman plot for the simulated data. The two panels in the first row are for Sample 1 versus Sample 2, where all SNPs are genetically homogeneous. In the modified Bland–Altman plot, the relative difference is within 0.02 for the majority of the points (i.e., 93.5%). The two panels in the second row are for Sample 1 versus Sample 3, and no SNP is expected to be genetically homogeneous. In the modified Bland–Altman plot, 62.2% of the SNPs have an absolute relative difference beyond 0.02.

The V‐V plot and the modified Bland–Altman plot for comparison of Sample 1 versus Samples 2 and 3 are shown in Figure 1. Given the way these summary statistics are generated, we expect that every SNP is genetically homogeneous between Samples 1 and 2, but not so between Samples 1 and 3. Indeed, the V‐V plots in Figure 1 verify these expectations. In the modified Bland–Altman plot for sample 1 versus sample 2, the relative difference is within 0.02 for the majority of the points (i.e., 93.5%). Using the criterion, 61.9% of the SNPs in the Sample 1 versus Sample 3 comparison are not homogeneous. It is interesting that, for two real data samples that come from the same population (i.e., BMI‐UKBB‐1 and BMI‐UKBB‐2) to be shown in Section 4, the relative differences are all within 0.015, which is 25% smaller than the 0.02 threshold suggested by this simulation study. Further justification of using 0.02 as the exclusion criterion is provided later in this section.

**FIGURE 2:**
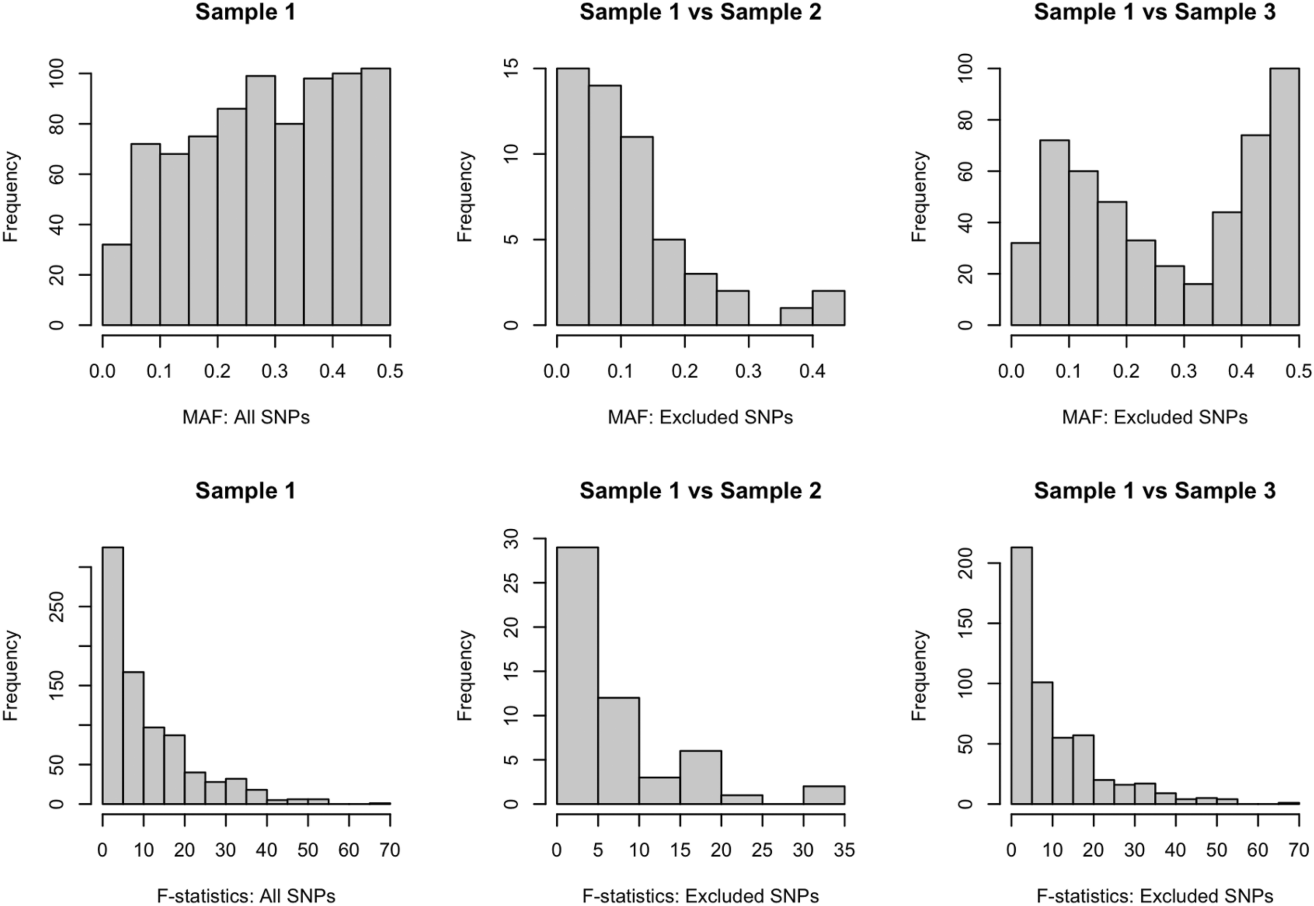
The histograms of MAF and F‐statistics for the same simulated data described in Figure 1. They show the distribution of the excluded SNPs in terms of MAF and F‐statistics. SNPs are excluded if the absolute relative difference between two samples is beyond 0.02.

One reviewer wondered whether instrumental strength or allele frequency has an effect on a SNP being excluded. Histograms of these metrics are shown in Figure 2. Compared with their frequency distribution, excluded SNPs with MAF close to 0 tend to have larger absolute relative differences, and such SNPs tend to have smaller F‐statistics.

We investigated the effects of several design choices on the proportion of excluded SNPs, including different exclusion thresholds (i.e., absolute relative differences larger than 0.01, 0.02, or 0.03), different sample sizes (10 000 or 5000), and different numbers of SNPs (812, 400, or 200). In the latter two cases, the 400 or 200 SNPs are randomly selected from the full set of 812 available SNPs. To further assess robustness, we also consider an endogenous covariate for Samples 1, 2, and 3, respectively,

$$\begin{array}{cc} x_{1} & =0.01\overset{812}{\underset{j=1}{\sum }}z_{ij}+N(0,1),\;x_{2}=0.02\overset{812}{\underset{j=1}{\sum }}z_{ij}+N(0,1),\\ \;x_{3} & =0.03\overset{812}{\underset{j=1}{\sum }}z_{ij}+N(0,1). \end{array}$$

The $R^{2}$s computed from the simulated data are around 15%, 20%, 30%, respectively. The results of these simulations are summarized in Table 1.

**TABLE 1:** Simulated proportion of absolute relative difference $\left|(v_{j}^{\left(y\right)}-v_{j}^{\left(x\right)})/(v_{j}^{\left(y\right)}+v_{j}^{\left(x\right)})\right|$ larger than a threshold under different scenarios. Sample 1 and Sample 2 share the same allele frequencies. Allele frequencies between Samples 1 and 3 are different.

Threshold	Sample size	Covariate?	# of SNPs	Sample 1 versus Sample 2	Sample 1 versus Sample 3
0.01	10 000	No	812	0.255	0.807
			400	0.245	0.788
			200	0.245	0.820
		Yes	812	0.256	0.808
			400	0.268	0.793
			200	0.255	0.845
	5000	No	812	0.452	0.799
			400	0.438	0.828
			200	0.460	0.790
		Yes	812	0.451	0.797
			400	0.423	0.783
			200	0.440	0.810
0.02	10 000	No	812	0.065	0.619
			400	0.073	0.621
			200	0.075	0.696
		Yes	812	0.064	0.619
			400	0.075	0.600
			200	0.050	0.610
	5000	No	812	0.161	0.584
			400	0.153	0.595
			200	0.155	0.600
		Yes	812	0.160	0.583
			400	0.158	0.615
			200	0.195	0.590
0.03	10 000	No	812	0.017	0.373
			400	0.018	0.325
			200	0.010	0.355
		Yes	812	0.017	0.376
			400	0.018	0.363
			200	0.010	0.370
	5000	No	812	0.052	0.387
			400	0.043	0.440
			200	0.060	0.345
		Yes	812	0.052	0.383
			400	0.040	0.410
			200	0.070	0.400

Several notable observations emerge from Table 1. First, the inclusion of covariates in the GWASs has little impact on the proportion of SNPs that are excluded. Second, as expected, the number of SNPs included in the analysis does not have a systematic effect, as the 400 and 200 SNPs are randomly selected from the full set of 812. In contrast, the exclusion threshold and the sample size do affect the results. Interestingly, sample size influences the comparison between Samples 1 and 2, but not the comparison between Samples 1 and 3. In the latter case, it may be due to the fact that differences in allele frequencies dominate the effect of sample size. As anticipated, larger exclusion thresholds lead to smaller proportions of excluded SNPs. Overall, compared with thresholds of 0.01 and 0.03, a threshold of 0.02 appears to strike a favorable balance: the exclusion proportion is not excessively high for the Sample 1 versus Sample 2 comparison, while not being too low for the Sample 1 versus Sample 3 comparison.

We also conducted simulations for a binary trait. The trait is generated from a binomial distribution with the probability of being 1 equal to d/(1+d), where $d=\exp[\log(1/9)+\sum_{j=1}^{812}b_{j}z_{ij}]$. That is, the prevalence of the trait is 10% among the subjects whose genotypes are 0 at all the SNPs. The summary statistics at the 812 SNPs are obtained using univariable (marginal) logistic regression. The results are close to what is shown in Figure 1 and are omitted.

## 4 Real Data Analyses

In this section, we apply the V‐V plot and the modified Bland–Altman plot to several published MR analyses; some data are from existing R packages, and some are from existing original publications. The goal is to check whether there are instrument SNPs that display genetic heterogeneity between the exposure GWAS and the outcome GWAS that are used in these studies.

### 4.1 Data From R Packages

In this subsection, we use data that come from R packages mr.raps (version 0.4.1) and mr.divw (version 0.1.0). These data are used to demonstrate the performance of the RAPS method [15] and the dIVW method [16] developed by the authors of these R packages. We consider four pairs of GWASs to demonstrate the utility of the proposed plots.

#### 4.1.1 Description of Data

The summary statistics for the first pair of GWASs are from data frame bmi.bmi in the R package mr.raps. This data frame contains summary data from three GWASs on BMI. Two of them, that is, BMI‐UKBB‐1 and BMI‐UKBB‐2, are used here because they are known to be from the same population, that is, United Kingdom BioBank (UKBB). BMI‐UKBB‐1 uses half of the UKBB data, and BMI‐UKBB‐2 uses the other half. There are 812 SNPs and there are 234 070 individuals in each sample. In this example, genetic homogeneity between these two samples is expected at every SNP.

The summary statistics for the second pair of GWASs are from data frame bmi.sbp in the R package mr.raps. It contains data from two GWASs denoted by BMI‐MAL and SBP‐UKBB. BMI‐MAL is a GWAS on BMI from the Genetic Investigation of ANthropometric Traits (GIANT) consortium, where the summary statistics are calculated using 152 893 males. SBP‐UKBB is a GWAS on systolic blood pressure using the United Kingdom BioBank (UKBB) data where the sample size is 317 754. According to its website, the GIANT consortium “is a collaboration between investigators from many different groups, institutions, countries, and studies, and the results represent their combined efforts.” Some degree of genetic heterogeneity between GIANT and SBP‐UKBB is expected. The number of SNPs is 160.

The summary statistics for the other two pairs of GWASs are from the data frame bmi.cad in the R package mr.divw. According to its documentation, this dataset contains summary data from a GWAS on BMI in round 2 of the UK BioBank (sample size: 336 107), a GWAS for CAD from the CARDIoGRAMplusC4D consortium (sample size: 185 000), with genotype imputation using the 1000 Genome Project, and GWAS for BMI in the Japanese population (sample size: 173 430). There are 1119 SNPs. Two comparisons are conducted. One is between the UK BioBank BMI sample and the CARDIoGRAMplusC4D consortium. The other is between the Japanese data and the CARDIoGRAMplusC4D consortium.

A summary of the four GWASs used in these four pairs of GWASs is provided in Table 2. We note that, for the CAD study, the phenotype is binary.

**TABLE 2:** Summary of the data used in the five data examples. All datasets are available in R packages mr.raps and mr.divw. BMI‐MAL is a GWAS on BMI from GIANT consortium and is expected to be a mixture of several populations. BMI‐Japanese is a GWAS on BMI from Japanees population. All other GWASs are from European population [14].

R Package	Data frame	# of SNPs	Exposure GWAS (Sample size)	Outcome GWAS (Sample size)
mr.raps	bmi.bmi	812	BMI‐UKBB‐1 (234070)	BMI‐UKBB‐2 (234070)
mr.raps	bmi.sbp	160	BMI‐MAL (152893)	SBP‐UKBB (317754)
mr.divw	bmi.cad	1119	BMI‐UKBB (336107)	CARDIoGRAMplusC4D Consortium (185000)
mr.divw	bmi.cad	1119	BMI‐Japanese (173430)	CARDIoGRAMplusC4D Consortium (185000)

#### 4.1.2 V‐V Plots and Modified Bland–Altman Plots

**FIGURE 3:**
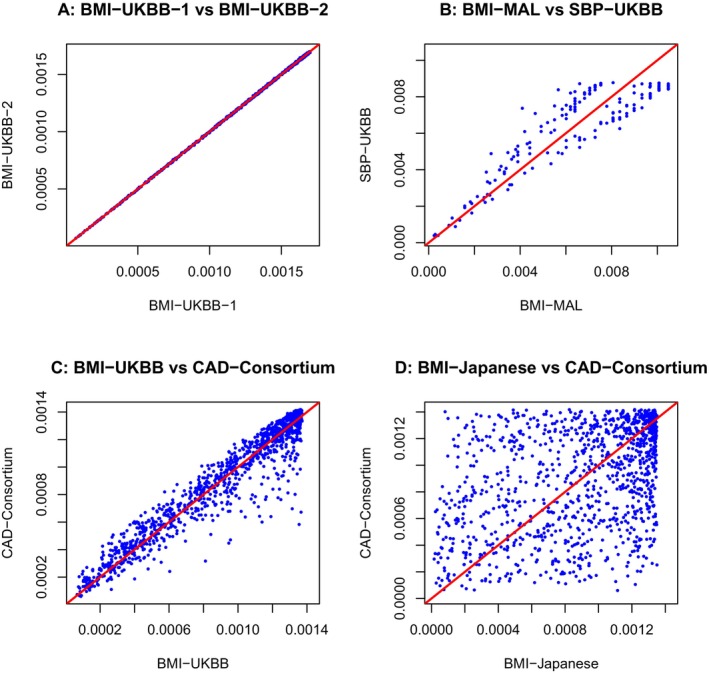
V‐V plots for datasets from the R packages mr.raps and mr.divw. The red line is the 45‐degree line going through the origin (0, 0). See the text for an explanation of each sample.

V‐V plots for the four pairs of GWASs are shown in Figure 3. As expected, the points in panel A follow a 45‐degree line going through point (0, 0), suggesting all SNPs are genetic homogeneous between BMI‐UKBB‐1 and BMI‐UKBB‐2. The points in Panel B of Figure 3 roughly follows the 45‐degree line but with many points away from this line, suggesting the presence of genetic heterogeneity at these SNPs between BMI‐MAL and BMI‐UKBB. Panel C is the plot for the UK BioBank BMI study and the CAD study. The points are somewhat scattered around the 45‐degree line, suggesting genetic heterogeneity between these two samples. As a matter of fact, the subjects for this study are mainly European, South Asian, and East Asian [17]. Panel D shows the plot for the CAD study and the Japanese BMI study. The lack of a pattern among the points in this plot suggests the existence of strong genetic heterogeneity between the Japanese BMI study and the CAD study, which is expected. It is interesting to see that the intercept in panel C is about the same as the intercept in panel D, suggesting the ratio of the variance of the disease status in the CAD study to the variance of the BMI are about the same even though BMI‐JPN and BMI‐UKBB are based on genetically different populations.

**FIGURE 4:**
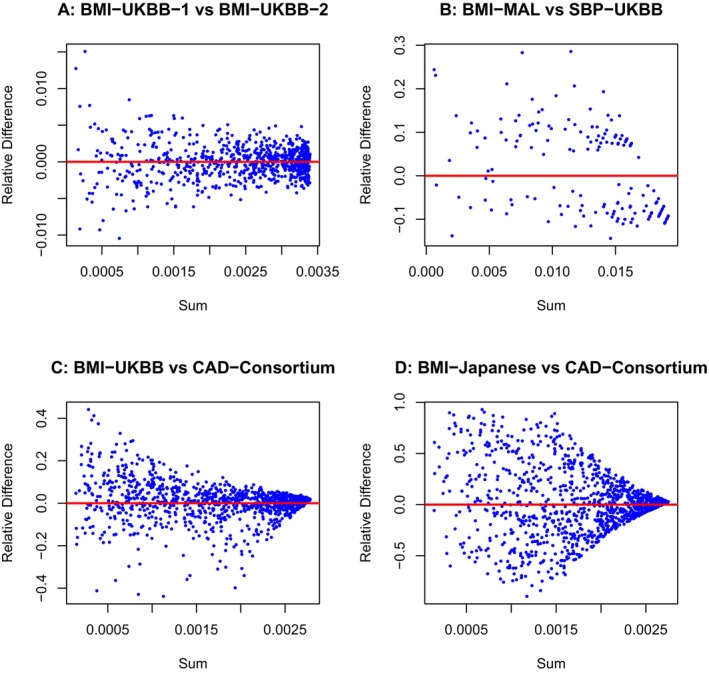
Modified Bland–Altman plots for datasets from the R packages mr.raps and mr.divw. The red line is horizontal with vertical intercept equal to 0.

To determine the SNPs that are genetically heterogeneous, the modified Bland–Altman plots for these four comparisons are presented in Figure 4. For the genetically identical samples shown in panel A, the relative differences are all within ±0.015=±1.5%, a value even smaller than the 0.02 threshold suggested by our simulation study. In comparison, many of the relative differences in the other three panels are beyond ±0.015, and they are not recommended to be included in a two‐sample summary‐data MR.

In summary, these plots demonstrate their usefulness in checking genetic homogeneity of SNPs between two samples.

#### 4.1.3 Sensitivity Analysis

To investigate the sensitivity of a two‐sample summary‐data MR to the inclusion of genetic heterogeneous SNPs, different thresholds (i.e., 0.01, 0.015, 0.02, 0.05, 0.1, 0.2, and 1) on the relative differences $\left(v_{j}^{\left(y\right)}-v_{j}^{\left(x\right)})/(v_{j}^{\left(y\right)}+v_{j}^{\left(x\right)}\right)$ are adopted for two particular comparisons: bmi.sbp and bmi.cad. The first one was analyzed in Zhao et al. [15] and the second one was analyzed in Ye et al. [16]. For each threshold, three popular two‐sample summary‐data MR methods, that is, IVW, dIVW, and RAPS, are applied to the SNPs whose relative differences are below the threshold. Estimates and their standard errors (SEs) from these methods are obtained using the R packages MendelianRandomization (for IVW and dIVW) and mr.raps (for RAPS). Note that using threshold value 1 does not exclude any SNPs since the relative difference does not exceed 1 in magnitude. Results of this sensitivity analysis are presented in Table 3.

**TABLE 3:** Sensitivity analysis of three popular two‐sample summary‐data MR methods to the use of different threshold values on the relative difference $\left(v_{j}^{\left(y\right)}-v_{j}^{\left(x\right)})/(v_{j}^{\left(y\right)}+v_{j}^{\left(x\right)}\right)$.

Data frame	Threshold	Number of instrument SNPs	IVW	dIVW	RAPS
bmi.sbp ^a^	0.01	1	−2.704 (4.964)	0.515 (1.153)	−2.704 (8.605)
	0.015	4	−2.914 (2.010)	−3.487 (1.026)	−3.060 (2.191)
	0.02	4	−2.914 (2.010)	−3.487 (1.026)	−3.060 (2.191)
	0.05	19	−0.155 (0.586)	−0.181 (0.202)	1.233 (0.507)^c^
	0.1	112	0.295 (0.123)	0.326 (0.065)	0.333 (0.123)
	0.2	152	0.310 (0.113)	0.352 (0.063)	0.377 (0.120)
	1	160	0.317 (0.111)	0.363 (0.063)	0.388 (0.118)
bmi.cad ^b^	0.01	199	0.437 (0.141)	0.502 (0.137)	0.486 (0.169)
	0.015	281	0.342 (0.123)	0.399 (0.122)	0.387 (0.147)
	0.02	364	0.248 (0.109)	0.290 (0.108)	0.290 (0.131)
	0.05	722	0.316 (0.072)	0.365 (0.073)	0.390 (0.085)
	0.1	916	0.332 (0.060)	0.379 (0.061)	0.392 (0.071)
	0.2	1064	0.313 (0.057)	0.360 (0.059)	0.377 (0.069)
	1	1119	0.315 (0.057)	0.365 (0.058)	0.382 (0.068)

^a^ This is the example data used by Zhao et al. [15]. A selection GWAS was used to select instrument SNPs in their analysis. The data set for the exposure GWAS is BMI‐MAL, and the data set for the outcome GWAS is CARDIoGRAMplusC4D Consortium (Table 2).

^b^ This is the example data used by Ye et al. [16]. A selection GWAS was used to select instrument SNPs in their analysis. The data set for the exposure GWAS is BMI‐UKBB, and the data set for the outcome GWAS is CARDIoGRAMplusC4D Consortium (Table 2).

^c^ According to the R output, the estimate may not be unique.

Many SNPs are excluded when a threshold 0.02 on the relative difference is used. For the bmi.sbp data, the number of included SNPs drops from 160 to merely 4, representing a 97.5% drop. For the bmi.cad data, the drop in the number of SNPs is 67.5% (from 1119 to 364).

Table 3 indicates that, no matter which MR method is used, exclusion of SNPs has a large effect on the estimate and its standard error for the bmi.sbp data. There is even a change in the direction of the estimate. This may be due to the relatively small number of SNPs, which is 160, to begin with. The bmi.cad data set has more SNPs (i.e., 1119 SNPs). When excluding SNPs, the effect estimate tends to be larger regardless of the method that is used.

We note that, in an MR analysis, the SNPs that remain are subject to another selection in order to choose SNPs that are instruments. That is, not all remaining SNPs are used for MR. Such selection for instrument SNPs is an issue different from checking genetic homogeneity. In both Zhao et al. [15] and Ye et al. [16], a third independent GWAS is used for instrument SNPs selection. The MR results presented in Table 3 use all remaining SNPs and no further selection is used for choosing instrument SNPs.

### 4.2 Data From Existing Original Research

We now examine two datasets employed in MR analyses by researchers in applied disciplines seeking to generate scientific insights. In both cases, the instrumental SNPs are selected directly from the same exposure GWAS used for MR inference, rather than from an independent GWAS‐a strategy adopted in other studies to mitigate bias [15]. As a result, these published MR analyses are susceptible to the “winner's curse,” whereby effect sizes are subject to selection bias [18, 19].

#### 4.2.1 Acetone and Essential Hypertension

**FIGURE 5:**
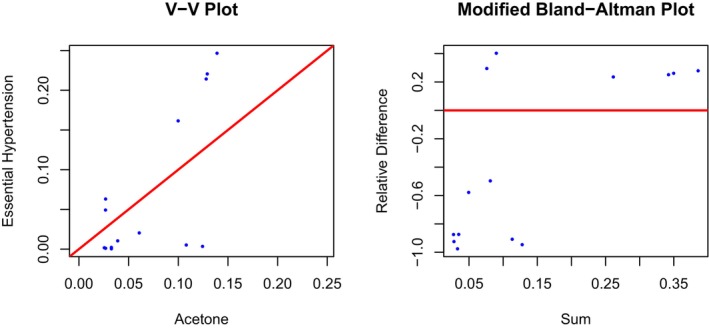
V‐V plot and modified Bland–Altman plot for summary statistics from GWASs on acetone and essential hypertension. There are 14 instrument SNPs.

The first data is from a MR analysis of the causal effect of acetone, a circulating metabolic biomarker, on essential hypertension [20]. Summary statistics for the acetone GWAS are publicly available through the NHGRI‐EBI GWAS catalogue (GCST90301942, acetone; 4435 East Asian, 11 340 South Asian, and 114 536 European). Summary statistics for the essential hypertension GWAS is from FinnGen Study [21] (release 12; phenocode: I9_HYPTENSESS; 345 634 controls and 132 515 cases according to file finngen_R12_pheno_n.tsv which is available through the website https://finngen.gitbook.io/documentation/data‐download). Similar to the MR analysis in Karjalainen et al. [20], SNPs strongly associated with acetone (i.e., p‐value $\leq 5\times 10^{-8}$) are selected and the summary statistics from the two GWASs are merged by chromosome and base‐pair position since the acetone GWAS does not contain SNP rs‐names. The V‐V plot and the modified Bland–Altman plot for these two samples are presented in Figure 5. There are 14 SNPs in these two plots. Based on the modified Bland–Altman plot, all the relative differences are much greater than 0.02 (in absolute value), suggesting none of the 14 SNPs are homogeneous between the two samples.

#### 4.2.2 Free Cholesterol Levels in Very Large HDL and Alzheimer's Disease

The summary statistics for the second data come from an MR study of the causal effect of blood metabolites that were previously associated with midlife cognition on Alzheimer's disease (AD) [22]. This study uses metabolite data from Kettunen et al. [23] and clinically diagnosed AD data from Knuckle et al. [24]. Full GWAS summary statistics on free cholesterol levels in very large high‐density lipoproteins (HDL) [23] are publicly available the from the NHGRI‐EBI Catalog of human GWASs (https://www.ebi.ac.uk/gwas/studies/, study accession: GCST90132680). The summary statistics for AD is made publicly available by International Genomics of Alzheimer's Project (IGAP) at https://archive.niagads.org/datasets/ng00075 (NG00075‐IGAP Rare Variant Summary Statistics Kungle et al. (2019)). The IGAP summary statistics do not contain information on allele frequency. Like the MR study previously presented, this study also selects SNPs strongly associated with the exposure (i.e., p‐value $\leq 5\times 10^{-8}$) as instrument SNPs. This MR study uses only the IGAP stage 1 data (see the next paragraph). The total number of instrument SNPs is 98, after merging with summary statistics for IGAP stage 1 data.

IGAP [24] is a large three‐stage study based upon genome‐wide association studies (GWAS) on individuals of European ancestry. In stage 1, IGAP used genotyped and imputed data on 11 480 632 SNPs to meta‐analyse GWAS datasets consisting of 21 982 Alzheimer's disease cases and 41 944 cognitively normal controls from four consortia: The Alzheimer Disease Genetics Consortium (ADGC); The European Alzheimer's disease Initiative (EADI); The Cohorts for Heart and Aging Research in Genomic Epidemiology Consortium (CHARGE); and The Genetic and Environmental Risk in AD Consortium Genetic and Environmental Risk in AD/Defining Genetic, Polygenic and Environmental Risk for Alzheimer's Disease Consortium (GERAD/PERADES). In stage 2, 11 632 SNPs were genotyped and tested for association in an independent set of 8362 Alzheimer's disease cases and 10 483 controls. Meta‐analysis of variants selected for analysis in stage 3A (n = 11 666) or stage 3B (n = 30 511) samples brought the final sample to 35 274 clinical and autopsy‐documented Alzheimer's disease cases and 59 163 controls.

**FIGURE 6:**
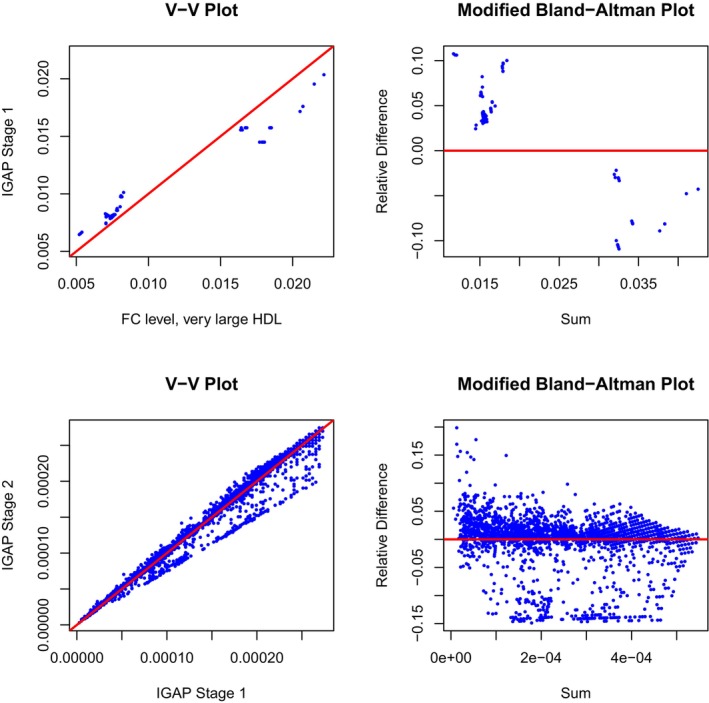
V‐V plots and modified Bland–Altman plots using summary statistics from GWASs on (first row) free cholesterol levels in very large HDL and Alzheimer's disease (98 instrument SNPs) and (second row) IGAP stage 1 and IGAP stage 2 (5843 SNPs).

The V‐V plot and the modified Bland–Altman plot based on the 98 instrument SNPs are presented in Figure 6. It the modified Bland–Altman plot, the relative differences for all 98 SNPs are greater than 0.02 (in absolute value, the smallest relative difference is 0.021). That is, these SNPs are unlikely to be homogeneous.

Since the IGAP data have two stages and samples for both stages are from individuals of European ancestry, it would be interesting to check whether SNPs are homogeneous between these two stages. The V‐V plot and the modified Bland–Altman plot are also presented in Figure 6. There are 5842 SNPs that are common to these two samples. It is surprising that many of them are not homogeneous: 1103 (18.9%) of them have a relative difference larger than 0.02 in absolute value. This result seems to be consistent with the common knowledge that there exists population heterogeneity among individuals of European ancestry.

## 5 Conclusion

We have proposed a graphical method to check the genetic homogeneity of a SNP between any two GWASs using only summary statistics. These two GWASs do not need to be independent, and the SNPs do not need to be in linkage equilibrium. However, if the SNPs are in linkage disequilibrium (LD), the threshold for the relative difference, which is 0.02 in magnitude, used for the modified Bland–Altman plot would no longer be applicable, as it is determined by a simulation study assuming linkage equilibrium.

This study is grounded in a fundamental statistical observation: in marginal regression at a SNP, the sum of the regression sum of squares (SSR) and the residual sum of squares (SSE) equals the total sum of squares (SST). Importantly, SST remains invariant across SNPs. This invariance implies that, for the purpose of comparing SNP‐specific variances between two samples, it is sufficient to know the relative magnitudes of the $v_{j}$ terms.

As noted by a reviewer, the proposed method is applicable exclusively to population‐based samples. Specifically, it assumes that both the exposure GWAS and the outcome GWAS are derived from representative population cohorts. If either GWAS is based on a selective sample, such as a case‐control study, the method may not yield valid or reliable results.

In this report, we focused on checking genetic homogeneity between two GWASs used for MR analysis. However, the proposed method can be used in other contexts that need genetic homogeneity. For instance, for SNP heritability estimation, LD score regression needs LD scores from an ancestry‐matched database [25].

## Funding

This work was supported by the National Institute of Environmental Health Sciences (Grant No. P30 ES005605). GERAD/PERADES was supported by the Medical Research Council (Grant n° 503480), Alzheimer's Research UK (Grant n° 503176), the Wellcome Trust (Grant n° 082604/2/07/Z), and the German Federal Ministry of Education and Research (BMBF): Competence Network Dementia (CND) grant n° 01GI0102, 01GI0711, 01GI0420. CHARGE was partly supported by the NIH/NIA grant R01 AG033193 and the NIA AG081220 and AGES contract N01‐AG‐12100, the NHLBI grant R01 HL105756, the Icelandic Heart Association, and the Erasmus Medical Center and Erasmus University. ADGC was supported by the NIH/NIA grants: U01 AG032984, U24 AG021886, U01 AG016976, and the Alzheimer's Association grant ADGC‐10‐196728.

## Conflicts of Interest

The authors declare no conflicts of interest.

## Data Availability

Data used in Section 4.1 are publicly available from R packages mr.raps (version 0.4.1) and mr.divw (version 0.1.0). Summary statistics for the acetone GWAS are publicly available through the NHGRI‐EBI GWAS catalog (GCST90301942). Summary statistics for the essential hypertension GWAS are from the FinnGen study [21] (release 12; phenocode: I9_HYPTENSESS). Full GWAS summary statistics on free cholesterol levels in very large high‐density lipoproteins (HDL) are publicly available from the NHGRI‐EBI Catalog of human GWASs (https://www.ebi.ac.uk/gwas/studies/, study accession: GCST90132680). The summary statistics for AD is made publicly available by International Genomics of Alzheimer's Project (IGAP) at https://archive.niagads.org/datasets/ng00075 (NG00075). The proposed V‐V plot and the modified Bland–Altman plot have been implemented in R package iGasso.

## Appendix A Approximation for a Logistic Model GWAS

For a logistic model GWAS, Equation (3) holds approximately to a multiplying factor. Below is a proof.

We use the same notation as in Subsection 3.1. But now the trait w takes only two values. Without loss of generality, these two values are denoted by 0 and 1. Let p=Pr(w=1). Consider the following marginal logistic regression model at a SNP with genotype score z:

$$\text{logit}(p)=\beta_{0}+\beta_{1}z.$$

It is well known that the maximum likelihood estimates of $\beta_{0}$ and $\beta_{1}$ satisfy

$$\overset{n}{\underset{i=1}{\sum }}(w_{i}-p_{i})=0,\;\;\overset{n}{\underset{i=1}{\sum }}(w_{i}-p_{i})z_{i}=0,$$

where n is the number of subjects. These equations are of the same form as the equations that determine the coefficients in a linear regression that regresses $w_{i}$ over $p_{i}$. However,

$$p_{i}=\frac{\exp(\beta_{0}+\beta_{1}z_{i})}{1+\exp(\beta_{0}+\beta_{1}z_{i})}$$

is a nonlinear function of $z_{i}$. To approximate $p_{i}$ by a linear model, say $p_{i}=\gamma_{0}+\gamma_{1}z_{i}$, a Talor series expansion around 0 is used. Define

$${\hat{\gamma }}_{0}=\frac{\exp(\beta_{0})}{1+\exp(\beta_{0})}.$$

We have

$${\left.p_{i}\right|}_{z_{i}=0}={\hat{\gamma }}_{0},\;\;{\left.\frac{\partial p_{i}}{\partial z_{i}}\right|}_{z_{i}=0}={\hat{\gamma }}_{0}(1-{\hat{\gamma }}_{0})\beta_{1}.$$

So an approximation of $\gamma_{1}$ is ${\hat{\gamma }}_{1}={\hat{\gamma }}_{0}(1-{\hat{\gamma }}_{0})\beta_{1}$, which is estimated by ${\hat{\gamma }}_{0}(1-{\hat{\gamma }}_{0}){\hat{\beta }}_{1}$ with a variance equal to ${\left[{\hat{\gamma }}_{0}(1-{\hat{\gamma }}_{0})\right]}^{2}\mathrm{Var}(\beta_{1})$. Equation (3) holds up to a multiplying factor $1/{\left[{\hat{\gamma }}_{0}(1-{\hat{\gamma }}_{0})\right]}^{2}$ because

$$\begin{array}{cc} v & =\frac{1}{{\hat{\gamma }}_{1}^{2}+(n-2-k)\mathrm{Var}({\hat{\gamma }}_{1})}\\ & =\frac{1}{{\left[{\hat{\gamma }}_{0}(1-{\hat{\gamma }}_{0})\right]}^{2}}\cdot \frac{1}{{\hat{\beta }}_{1}^{2}+(n-2-k)\mathrm{Var}({\hat{\beta }}_{1})}. \end{array}$$

A multiplying factor does not affect the V‐V plot and the modified Bland–Altman plot as both plots are based on the relative size of v with respect to the vs at other SNPs.

A similar result holds for Poisson model GWAS. The proof is similar to the case of logistic model GWAS and is omitted.
